# Ethical Perspectives on Suicide and End-of-Life from Aristotelian Virtue Ethics to Modern Psychoanalytic Approaches: a critical appraisal and review of literature

**DOI:** 10.1192/j.eurpsy.2026.11895

**Published:** 2026-07-31

**Authors:** J. Eberlein, M. Chorianopoulou, C. Dokos

**Affiliations:** 1 St. Josef Psychiatric Hospital AMEOS, Oberhausen, Germany; 2 School of Philosophy, National and Kapodistrian University of Athens, Athens, Greece; 3 Department of Acute and Emergency Medicine, Klinikum Luedenscheid, Luedenscheid, Germany

## Abstract

**Introduction:**

Ethical debates on suicide and end-of-life choices have evolved significantly over time. Aristotle’s virtue ethics emphasized that human flourishing (*eudaimonia*) depended on moral character as well as sound judgment, including decisions about life and death (MacIntyre A. *After Virtue.* 3rd ed. Notre Dame: University of Notre Dame Press; 2007). However, modern debates are more focused on autonomy, beneficence, and the clinician’s duty towards preserving life, particularly within psychiatric settings.

**Objectives:**

To critically appraise historical and contemporary ethical frameworks on suicide and end-of-life decisions, with particular emphasis on the transition from virtue ethics to modern psychoanalytic approaches.

**Methods:**

A conceptual literature review was conducted, analyzing philosophical, psychiatric, and ethical sources. Key themes included virtue ethics, principles of biomedical ethics, psychodynamic theories of suicidality, and relational models of autonomy.

**Results:**

The review shows a continuous shift in ethical reasoning about suicide and end-of-life care, moving from character-based to principle-driven and relational approaches. Virtue ethics emphasized the importance of moral character and the pursuit of a meaningful life within the community, whereas modern frameworks focus on autonomy while also incorporating relational dimensions (Beauchamp TL, Childress JF. *Principles of Biomedical Ethics.* 8th ed. Oxford: Oxford University Press; 2019). Psychodynamic perspectives enrich this discussion by highlighting the influence of hopelessness, guilt, and interpersonal dynamics in suicidality (Maltsberger JT. *The descent into suicide.* Int J Psychoanal. 2004;85(3):653–670). More recent research promotes relational autonomy, emphasizing family involvement and community values in guiding end-of-life decisions (Dove ES, Kelly SE. *BMC Med Ethics* 2015;16:45). Together, these perspectives demonstrate how ethical analysis has expanded from the individual to the relational and societal level, offering clinicians a more comprehensive framework for practice.

**Conclusions:**

The evolution from Aristotelian virtue ethics to psychoanalytic and relational approaches marks a shift towards a more nuanced perspective on suicide and end-of-life care. Thus, integrating autonomy, beneficence, and relational ethics provides clinicians with a balanced and compassionate framework for practice.

**Disclosure of Interest:**

None Declared

